# Creative solutions to extraordinary challenges in clinical trials: methodology of a phase III trial of azithromycin and chloroquine fixed-dose combination in pregnant women in Africa

**DOI:** 10.1186/1475-2875-12-122

**Published:** 2013-04-11

**Authors:** Richa S Chandra, John Orazem, David Ubben, Stephan Duparc, Jeffery Robbins, Pol Vandenbroucke

**Affiliations:** 1 Medical Development Group, Emerging Markets Business Unit, Pfizer Inc, 445 Eastern Point Road, MS 8260-2107, Groton, CT, 06320, USA; 2 Medicines for Malaria Venture, 20, rte de Pré-Bois, Geneva 15, 1215, Switzerland

**Keywords:** Azithromycin, Chloroquine, Intermittent preventive treatment, IPTp, AZCQ, Adaptive design

## Abstract

**Background:**

Malaria in pregnancy is one of the most common preventable causes of maternal and neonatal morbidity and mortality in sub-Saharan Africa. To prevent its adverse effects, such as maternal anaemia, placental parasitaemia and low birth weight (LBW) neonates, the World Health Organization recommends effective malaria case management, use of insecticide-treated bed nets and intermittent preventive therapy in pregnancy (IPTp). Sulphadoxine-pyrimethamine (SP) has been the standard for IPTp in several countries, but parasite resistance to SP is growing. Therefore, new IPTp therapies are urgently needed. One candidate being evaluated for IPTp is a fixed-dose combination of azithromycin and chloroquine (AZCQ). This paper describes the challenges and the innovative solutions implemented in designing and conducting a pivotal AZCQ-IPTp trial, sponsored by Pfizer Inc and co-funded by Pfizer Inc and the Medicines for Malaria Venture.

**Methods:**

The AZCQ-IPTp pivotal trial is a multicentre, multicountry, phase III, open-label, randomized superiority study of AZCQ-IPTp *versus* SP-IPTp in pregnant women of sub-Saharan Africa. The trial was designed to meet stringent regulatory agency scientific advice and IPTp policy makers’ recommendations, and incorporates an innovative adaptive design to manage programme risk, maintain the operating characteristics of the study and optimize resources. The trial’s novel composite primary endpoint is the proportion of participants with a suboptimal pregnancy outcome (abortion [≤28 weeks], stillbirths [>28 weeks], premature [<37 weeks] deliveries, LBW [<2,500 g] live neonates, missing neonatal birth weight data or loss to follow-up). The study employs a prospective group sequential design with three unblinded analyses when 50%, 70% and 100% of participants achieve the primary endpoint; the study team will remain blinded to the analyses until after the completion of the study. The number of participants randomized will be adaptive, based on the blinded review of the observed pooled primary endpoint data across the two treatment arms, when approximately 1,000 participants complete the primary endpoint assessments.

**Results:**

This study is ongoing and expected to complete in 2014.

**Conclusion:**

This report describes the unique challenges and innovative solutions implemented in designing and conducting this pivotal AZCQ-IPTp trial, which may serve as a prototype for future IPTp and other studies involving similar conditions.

## Background

Malaria in pregnancy (MiP) is one of the most common preventable causes of maternal and neonatal mortality and morbidity in sub-Saharan Africa. In 2007, 54.5 million pregnancies were at risk in stable and high *Plasmodium falciparum* transmission areas of sub-Saharan Africa, and another 70.5 million in areas with *Plasmodium vivax* only or low transmission [1].

The common adverse outcomes of malaria infection in pregnant women, with considerable acquired immunity, living in areas of high and stable *P. falciparum* malaria transmission, include maternal anaemia, placental parasitaemia and low birth weight (LBW; weighing <2,500 g) neonates [2-5]. LBW is primarily a result of placental infection and consequent intra-uterine growth retardation [3]. Primigravidae and secundigravidae women have higher prevalence of maternal malaria infection (peripheral and placental parasitaemia) and their neonates are at higher risk of LBW [4]. LBW has been associated with an increased risk of morbidity and mortality [6-8]. Intermittent preventive therapy in pregnancy (IPTp) is one of the key strategies recommended by the World Health Organization (WHO) Global Malaria Programme (GMP) for malaria control in areas of stable and high malaria transmission where many pregnant women with malaria infection remain asymptomatic [9]. The WHO’s recommended strategies include both curative (effective case management of malaria and anaemia) and preventive (insecticide-treated nets and intermittent preventive therapy) measures [10]. IPTp is expected to improve pregnancy outcomes by clearing asymptomatic parasitaemia, treating and/or preventing placental malaria and by preventing malaria and its adverse consequences. IPTp is based on the administration of a complete curative dosing regimen of (an) anti-malarial drug(s) at routine periodic antenatal visits during pregnancy and may provide protection over and above any protection provided by the use of bed nets [10].

Recent studies have shown that a wide range (27% to 58%) of women attending an antenatal care facility in sub-Saharan Africa were positive for *P. falciparum* parasitaemia [11-15]. One study estimated that universal coverage with IPTp would reduce all-cause neonatal mortality by 32% [16]. Based on the evidence of significant reduction in LBW neonates, placental malaria and maternal anaemia following the use of sulphadoxine-pyrimethamine (SP) in pregnant women, the WHO recommended SP as the standard of care for IPTp [17]. The 2012 WHO IPTp guidelines recommend that all pregnant women in areas of moderate and high malaria transmission receive SP-IPTp at each antenatal visit in the second and third trimester [17].

Several countries in sub-Saharan Africa had adopted SP-IPTp in their national malaria control programmes and antenatal care guidelines at the end of 2010 [18,19]. However, growing resistance to SP in recent years has become a cause for concern, especially in East and Southern Africa [20-22]. Hence, there is an urgent need for developing an effective replacement for SP-IPTp.

An ideal drug for IPTp needs to be safe and well tolerated in pregnancy, and efficacious in preventing the detrimental effects of malaria on the mother and the foetus. To limit the development of resistance, an IPTp drug should not be used as first-line treatment for symptomatic malaria [23]. Very few drug candidates or their combinations meet these criteria.

The Malaria in Pregnancy Consortium is currently investigating mefloquine (ClinicalTrials.gov identifier NCT00811421) and a dihydroartemisinin-piperaquine combination (NCT01669941 and NCT01231113) for IPTp. However, the use of the dihydroartemisinin-piperaquine combination could be limited by the known tolerability and neuropsychiatric issues associated with its use, especially in women [24]. The recent efforts of the Medicines for Malaria Venture (MMV) to develop a better tolerated enantiomer of mefloquine failed to demonstrate any advantage over the commercially available mefloquine [25].

A fixed-dose combination of azithromycin (AZ) and chloroquine (CQ), AZCQ, is currently under investigation for IPTp. The combination of AZ and CQ has demonstrated additive to synergistic activity against *P. falciparum in vitro*[26,27]. In a phase II clinical study in symptomatic uncomplicated *P. falciparum* malaria, AZ and CQ monotherapies were suboptimal in efficacy (33% and 27%, respectively); however, when AZ and CQ were co-administered, the efficacy was 97% [28]. Co-administration of AZ and CQ has demonstrated efficacy, safety and tolerability in two multi-country phase II/III clinical studies in the treatment of symptomatic uncomplicated *P. falciparum* malaria in adults in sub-Saharan Africa [28,29]. AZ and CQ have been on the market for several years and have extensive safety experience in adults, children and pregnant women. Both AZ and CQ have been widely used in all trimesters of pregnancy and are considered safe in pregnant women as individual agents [30]. The activity of AZ against common sexually transmitted infections (STIs) including gonorrhoea and chlamydial infections [30,31] could be an added benefit in improving pregnancy outcomes.

IPTp is recommended for high and stable malaria transmission regions, primarily in sub-Saharan Africa. To ensure review by a well-established regulatory agency, before seeking African regulatory approvals, the regulatory dossier for the AZCQ-IPTp programme will be submitted to the European Medicines Agency (EMA) under Article 58 for a Committee for Medicinal Products for Human Use (CHMP) Scientific Opinion. Article 58 is a new mechanism for products developed for countries outside the European Union for indications considered of public health interest by the WHO [32]. The EMA evaluates such dossiers in cooperation with the WHO for safety, efficacy, quality and risk-benefit for the populations for which the product is intended [32]. The EMA review and opinion will be important for subsequent review and approvals from African regulatory agencies and for the inclusion of AZCQ in WHO and African national policy guidelines to ensure access and delivery to pregnant women in areas of high malaria transmission.

This article describes the challenges in designing and conducting a phase III IPTp clinical trial and the innovative approaches being used to overcome these challenges.

## Methods

### Methodological issues in designing IPTp clinical trials

#### Absence of regulatory guidance

While SP-IPTp is included in policy guidelines for several countries in Africa, it has never been evaluated by any well-established regulatory bodies that review new drug applications, such as the EMA or US Food and Drug Administration. No regulatory guidance document is available on how to evaluate an investigational treatment for IPTp. Hence, from a regulatory perspective, IPTp is an unprecedented indication. To be accessible to the target population, a new IPTp drug would require approval by the national regulatory agencies and inclusion in the policy guidelines of the WHO and the National Malaria Control and Reproductive Health Programs. The standard IPTp trial designs used for the evaluation of SP-IPTp are not consistent with general clinical trial guidance from well-established regulatory authorities.

#### Primary endpoint definition

When designing the study, a review of the literature between 1990 through 2008 indicated the use of different endpoints as part of the primary endpoint, the three key endpoints being LBW, maternal anaemia and placental malaria. Moreover, there was no consistency in how these endpoints were defined. The WHO/GMP recommends LBW as an endpoint since it appears to correlate with child survival [10]. Most recent IPTp studies to date have used LBW as the primary endpoint; other trials have used severe anaemia or placental parasitaemia as the primary endpoint. While LBW seems to be widely accepted as the primary endpoint among the malaria research community, it has some limitations from a regulatory perspective as described in the paragraph below.

The LBW endpoint recommended by the WHO and used in several recently published IPTp studies is defined as the proportion of study participants with LBW (<2,500 g) live singleton neonates. The analysis of a LBW endpoint in these studies excludes participants with other suboptimal pregnancy outcomes, such as miscarriages, stillbirths and premature deliveries, and also excludes participants with missing neonatal birth weight data or participants who were lost to follow-up. Although not always clearly specified in IPTp clinical trials, the percentage of participants with incomplete information, such as missing birth weight data or those lost to follow-up, varies widely, with ranges between approximately 5% to 66% in trials that reported this information [33-35]. Regulatory agencies take a conservative approach and suggest inclusion of all those participants listed above in the primary endpoint analysis and categorize them as “failures” per data analyses guidelines from the EMA [36]. As a consequence, participants with any suboptimal pregnancy outcome or missing data, including the unknown outcome of pregnancy, absence of known birth weight and lost to follow-up are considered “failures.”

#### Sample size determination

Typically, sample size is calculated taking into consideration the estimated true effect size and the true incidence of the primary endpoint in the control group population. Since none of the IPTp studies to date have used the composite endpoint of suboptimal pregnancy outcomes, it is difficult to get a reliable estimate of the true incidence of this endpoint in participants on the active control SP arm. Moreover, even the incidence of the commonly used primary endpoint of LBW with SP-IPTp from the randomized controlled clinical trials reported in the literature over the last 10 years varies widely, from 8% to 24%, depending upon factors such as the gravidity (e.g. primigravida vs. multigravidae) and human immunodeficiency virus status of participants included in the study, definition of LBW endpoint (within 24 hours or within a week of delivery), concurrent use of insecticide-treated bed nets and other interventions, SP resistance status of parasites, region where the study was conducted, timing of the study, sample size and power of the study [37-39].

#### Effect size

A meta-analysis that included nine clinical trials conducted between 1996 and 2006 demonstrated that SP-IPTp reduced placental malaria, LBW and maternal anaemia [20]. Five of these trials compared SP-IPTp with placebo or case management and one study included the use of insecticide-treated nets. In this study, AZCQ-IPTp is being compared with SP-IPTp in pregnant women. Per the current standard of care, both treatment arms in the study are using insecticide-treated bed nets; consequently, the preventive effect of bed nets may further narrow the gap between the two treatment arms and result in a lower than expected effect size. Moreover, the effect size of SP-IPTp for the composite endpoint of suboptimal pregnancy outcome is unknown since none of the IPTp studies to date have evaluated this endpoint. More specifically for AZCQ, the activity of AZ against common STIs could be additive towards improving pregnancy outcomes and may positively impact the treatment effect in comparison with SP-IPTp, but the magnitude of this effect is unknown.

#### Rescue medication for symptomatic malaria

While an IPTp regimen should prevent and lower the number of malaria episodes during the course of pregnancy, it is not aimed at completely eliminating them. Hence, the study participants who develop symptomatic malaria during the study period are treated with appropriate rescue anti-malarial medication, such as an artemisinin-based combination or quinine as per local standard of care guidelines. The anti-malarial effect of any such rescue treatment could potentially confound the IPTp effect of the study treatments.

#### Operational issues in conducting phase III IPTp trials in sub-Saharan Africa

There are several practical challenges in conducting an IPTp trial in sub-Saharan Africa. The suboptimal regional regulatory infrastructure to evaluate a novel treatment, inadequate clinical research infrastructure, inexperience in conducting clinical trials as per the International Conference on Harmonisation-Good Clinical Practice (ICH-GCP) guidelines and stringent regulatory standards of western agencies are some of the key issues that must be overcome before initiating any study. In designing the AZCQ-IPTp trial, these challenges had to be evaluated and measures put in place to address them.

## Results

### AZCQ-IPTp pivotal trial design and conduct

This trial is sponsored and conducted by Pfizer Inc, is co-funded by Pfizer Inc and the MMV and has an oversight by an independent external data monitoring committee (EDMC) with three-quarters membership from sub-Saharan Africa. The clinical, statistical and regulatory plans and pivotal study design were discussed at a scientific advice meeting with the Medicines and Healthcare products Regulatory Agency (MHRA) in the UK and its input was incorporated.

### Trial design

The AZCQ-IPTp pivotal trial is an on-going multi-centre, multi-country, phase III, open-label, randomized, comparative, superiority study of AZCQ-IPTp *versus* SP-IPTp in pregnant women in sub-Saharan Africa. The trial, initiated in 2010, has been designed to meet both regulatory and WHO/GMP IPTp guidelines and has an adaptive design to manage programme risk as well as to maintain the operating characteristics of the study, and to optimize timelines and resource utilization.

### Primary endpoint

The pivotal study incorporates the composite endpoint of a suboptimal pregnancy outcome as per MHRA Scientific Advice that takes into account all missing data including absence of birth weight information (e.g. in case of home deliveries) and any loss to follow-up. The composite endpoint for this study was defined as the proportion of participants with suboptimal pregnancy outcomes including abortion (≤28 weeks), stillbirths (>28 weeks), premature (<37 weeks) deliveries, LBW (<2,500 g) live neonates, missing neonatal birth weight data or loss to follow-up.

### Key secondary endpoints

The WHO/GMP recommended endpoint of occurrence at birth of LBW (<2,500 g) live neonates is a key secondary endpoint in this study. The evaluation of this endpoint may be included in any decisions regarding the potential incorporation of AZCQ in policy guidelines. Other key secondary endpoints include commonly used IPTp endpoints such as the occurrence of anaemia (haemoglobin <11 g/dL) or severe anaemia (haemoglobin <8 g/dL) at 36 to 38 weeks of gestation, occurrence of placental parasitaemia at delivery, occurrence of placental malaria as determined by histology and number of episodes of STIs per participant following the first dose. The STIs were diagnosed based on the participant’s clinical presentation at any time between the first dose of IPTp and delivery and/or laboratory test results for the specimens collected between weeks 36–38 of gestation.

### Sample size

The underlying true incidence of the primary endpoint in the control group of this study is unknown, but has a substantial impact on the required sample size. Based on the wide range of suboptimal pregnancy outcome rates, including LBW rates reported in recent IPTp studies, the total number of participants to be enrolled ranges from 2,602 to 5,044. To manage this uncertainty, the study plans to enrol up to 5,044 participants; the sample size will be finalized following the blinded review of the observed proportion of the primary endpoint of suboptimal pregnancy outcomes in the pooled data across the two groups after the first 1,000 participants have achieved the primary outcome (Table 1). For example, if the observed proportion of participants experiencing a suboptimal pregnancy outcome is found to be 0.30, then the total sample size will be 4,206. It should be noted that the total sample size required will be calculated by employing a group sequential design with the upper bound of each interval assumed to be the true underlying proportion for the SP group. Owing to the lack of reliable estimates in the literature for the primary endpoint rate in the control group, and given that the magnitude directly impacts the precision of an analysis, which in turn affects statistical power, the total number of participants randomized is planned to be adaptive.

**Table 1:** Sample size determination

**Observed pooled proportion for the primary endpoint at N = 1,000**	**Total sample size required**
≤0.28	5,044
>0.28 to ≤0.32	4,206
>0.32 to ≤0.36	3,552
>0.36 to ≤0.40	3,030
>0.40	2,602

The design assumed a 20% risk reduction in the primary endpoint (relative risk = 0.80, fixed-dose combination of azithromycin and chloroquine/sulphadoxine-pyrimethamine [AZCQ/SP]). A 23% risk reduction (relative risk = 0.77, AZCQ/SP) was assumed for low birth weight (key secondary endpoint).

The sample size estimation assumed a 20% risk reduction in the primary endpoint (relative risk = 0.80, AZCQ/SP). A 23% risk reduction (relative risk = 0.77, AZCQ/SP) was assumed for LBW (key secondary endpoint). The 20% target for the primary endpoint was considered a minimum clinically relevant treatment effect, and the 23% target for LBW is a consequence of the fact that LBW is a component of the primary endpoint.

### Interim and final analyses

The AZCQ-IPTp study employs a group sequential design with three unblinded comparative analyses conducted when 50%, 70% and 100% of participants complete the primary endpoint visit. Group sequential stopping rules will be used to stop the trial early for efficacy and futility (non-binding) at each of the two sequential analyses.

The overall alpha level will be one-sided 0.000625, controlled throughout the duration of the study using a pre-specified alpha spending function, with an approximate overall power of 90% for establishing the superiority of AZCQ over SP in a proportion of participants with suboptimal pregnancy outcomes (primary endpoint). The low alpha level of one-sided 0.000625 (equivalent to two-sided 0.00125) is a regulatory requirement when a single pivotal trial is used to demonstrate the same level of evidence as two independent pivotal trials, without actually performing a second trial. Secondary efficacy endpoints will use an overall alpha level of two-sided 0.05. Overall power, and equivalently the false negative rate, will be maintained by employing a pre-specified spending function.

Early stopping for positive efficacy will only occur if both the proportion of participants with suboptimal pregnancy outcomes (primary endpoint) and the proportion of participants with LBW (the first key secondary endpoint) are statistically significantly lower in the AZCQ group compared with the SP group, as evaluated by the relative risk (primary analysis). The exact significance level used will be derived from the alpha spending function at the time of each interim analysis. If the total sample size is 5,044, therefore, the sample size for the interim analysis is 2,522, and based on the spending function used, the significance level will be 0.0001 for the primary endpoint. Similarly, the exact significance level used for judging futility will be derived from the beta spending function at the time of each interim analysis. The sequential analyses will use unblinded treatment codes and be prepared by a group within Pfizer but external to the study team. The study team will remain blinded to the results. The EDMC will review the data every four months and recommend stopping or continuing the study based on the interim results, predefined alpha and beta spending functions and corresponding stopping boundaries and interim monitoring output. The membership of the EDMC and its charter, as well as the procedures for performing the interim analyses, were finalized and documented before the first interim analysis.

Owing to the adaptive nature of the sample size determination, operating characteristics of the study design in terms of power and Type I error rate were evaluated using simulation. Probabilities for early stopping for positive efficacy and futility at each of the interim analyses were evaluated as well. Type I error rates were simulated under the null hypothesis of no difference (relative risk = 1.00). Each scenario under the alternative hypothesis was run with 100,000 simulation iterations to estimate the power. One million iterations were used, under the null hypothesis, to determine the Type I error rate for each stated true proportion for the primary endpoint.

### Trial conduct

As the overall goal of the IPTp programme is to identify a potential replacement for SP, the study sites are located in areas with stable and high malaria transmission where SP-IPTp is included in national policy guidelines and SP resistance has emerged as an issue of concern. The participating countries include Benin, Kenya, Malawi, Tanzania and Uganda. Patient enrolment started in October 2010 and the study is estimated to complete in 2014.

### Site selection and capacity building

The trial sites were selected based on the level of malaria transmission, local SP resistance rates and rigorous pre-trial site assessments by the monitoring and audit teams. In addition, central laboratories in the region were audited before their selection to identify gaps and plan training for any deficiencies noted.

A number of processes have been put in place to ensure conduct of the trial according to ICH-GCP standards and smooth running of the day-to-day trial functions. The sites were assisted with infrastructure building as necessary for the trial conduct. A week-long investigator meeting was conducted in Johannesburg to train site study teams on ICH-GCP standards and Pfizer Standard Operating Procedures (SOPs) related to study conduct, protocol and laboratory assessments. Three-day hands-on ethics workshops on research in vulnerable populations were conducted in collaboration with the Steve Biko Centre of Bioethics (Johannesburg, South Africa) for investigators and ethics committee members for study sites. During the four weeks preceding the enrolment of the first participant, a five-day site initiation visit was conducted for each site to train site teams on the practical aspects of conducting the protocol as per ICH-GCP standards and Pfizer SOPs. The site teams are now adequately resourced to ensure compliance with the study protocol and adequate documentation is required for regulatory submissions. Each site team has engaged field workers for home visits to ensure compliance with dosing, implementation of insecticide-treated bed nets and adverse event monitoring.

The study has a robust monitoring plan. The first site visit is conducted within two weeks of the enrolment of the first patient and subsequent visits are conducted every three to six weeks, depending upon the enrolment rates and issues identified at previous visit(s). Each visit lasts about a week. Medical monitoring is conducted by Pfizer physicians on an on-going basis through a weekly review of enrolment and adverse event logs from each site, weekly teleconferences with the principal investigators for each site to review any clinical or safety issues and a monthly review of protocol deviations and blinded safety data from the Pfizer database populated from monitored (i.e. quality-controlled) case report forms. Site study teams are required to send information on emerging serious adverse events to Pfizer’s Safety Group within 24 hours of their first being aware of them. Emerging safety data also are reviewed monthly by a cross-functional clinical safety core group at Pfizer. In addition, cross-functional risk management and product benefit risk committees review the emerging data from all Pfizer-sponsored AZ studies and published literature to identify and evaluate any new safety signals or trends and update the benefit-risk assessment on an on-going basis. Initial weekly enrolment rates are deliberately kept low to give sites adequate time to get hands-on experience and achieve operational excellence. Each site is audited by an independent external audit agency early in the trial when approximately 10% of study participants are enrolled. In addition, internal quality review site visits are conducted every three months by Pfizer’s clinical operations lead. A root cause analysis is performed for any significant protocol deviations or issues identified during the monitoring, quality review or audit visits, site teams are retrained and corrective and preventive measures are put in place before sites can resume enrolment at optimum speed.

In addition, the EDMC, whose members include both international and African experts in both malaria and pregnancy, has been conducting a quarterly review of emerging unblinded safety data and also will review the interim analyses data. While the study is open-label, the study team remains blinded to the emerging aggregate safety data reviewed by the EDMC.

In the spirit of collaboration and to facilitate trial application review, face-to-face meetings with African national regulatory agencies were held prior to the submission of the clinical trial application. These meetings provided an overview of the programme and clinical study designs, and were especially important in view of the unprecedented nature of the IPTp indication as well as Pfizer’s intention to seek CHMP Article 58 scientific opinion.

## Discussion

Despite widespread use, IPTp is an unprecedented indication from the regulatory perspective and there is currently no regulatory guidance on how to conduct an IPTp trial for registration purposes. The published literature is not very informative in terms of designing a study with the rigour required for stringent regulatory review. To be accessible to pregnant women in sub-Saharan countries who would need it the most, a new IPTp regimen needs to be incorporated into the malaria control guidelines from the WHO/GMP, and following regulatory approval, in National Malaria Control and Reproductive Health Programs. Therefore, when Pfizer and the MMV initiated this AZCQ-IPTp trial, the team sought input from diverse stakeholders. There were several challenges in designing this trial, from selecting an appropriate study design, endpoints and sample size to the need for capacity building, including infrastructure and trained personnel for appropriate patient monitoring during the conduct of the study.

The innovative study design of the AZCQ-IPTp trial addresses the key challenges in designing and implementing this type of study. In addition to maintaining proper operating characteristics, the adaptive nature of the trial design will minimize any undue exposure of the pregnant women to study drugs by optimizing sample size estimation and through interim checks of the data. Given the unprecedented nature of the IPTp indication, early on during the planning phase, a scientific advice meeting was held with the MHRA.

Since the novel primary endpoint of suboptimal pregnancy outcomes may pose a challenge for policy makers while comparing data from other ongoing studies of investigational IPTp products and regimens, the AZCQ-IPTp study protocol also incorporates other commonly used IPTp endpoints as key secondary endpoints.

This study is being conducted according to ICH-GCP standards that required the implementation of a number of capacity-building initiatives such as infrastructure upgrades, training in ICH-GCP guidelines, ethics workshops and a robust monitoring plan to ensure all study personnel and procedures are in compliance with ICH-GCP guidelines, auditing of regional laboratories, quality assurance and operational team visits to rectify any deficiencies.

There are several limitations of this study. First, the primary endpoint of suboptimal pregnancy outcomes may be less sensitive than the endpoint of LBW recommended by the WHO and considered a standard primary endpoint. The composite endpoint of suboptimal pregnancy outcomes may have categories of endpoints that may not be affected by malaria or STI protective effect and hence may dilute the overall IPTp effect. As a result, the study may not be able to demonstrate superiority on the primary endpoint even though it may be positive on the endpoint of LBW, which is currently considered the more relevant endpoint from the policy perspective. In addition, the first 1,000 participants enrolled in the study predominantly include those from the first two to three sites or countries that started enrolling early, thereby having the potential to provide less accurate sample size estimation.

This paper describes the challenges and innovative solutions implemented in designing and conducting this pivotal AZCQ-IPTp trial. Conducting a study as per ICH-GCP standards for an unprecedented indication in the resource-constrained setting of sub-Saharan Africa requires significant capacity building, rigorous training and intense monitoring, all of which can be very resource consuming. The high cost, coupled with the corporate liability risk of conducting research in a vulnerable population in these settings, may be one reason why the pharmaceutical industry shies away from such much-needed trials. This trial is being conducted by Pfizer in collaboration with the MMV, a not-for-profit public private partnership foundation with a mission to reduce the burden of malaria in disease-endemic countries by discovering, developing and facilitating delivery of new, effective and affordable anti-malarial drugs. The MMV is not only providing financial support for the clinical trial but also provides invaluable scientific inputs and stakeholders engagement through a team of malaria experts and its external expert scientific advisory committee. Public-private partnerships, such as the Pfizer-MMV partnership, are one way that the pharmaceutical industry may contribute to the successful development of new medicines for vulnerable populations in low-income countries. The design and conduct of this study may serve as a prototype for future IPTp and other studies where similar conditions exist.

## Abbreviations

AZ: Azithromycin; AZCQ: Fixed-dose combination of azithromycin and chloroquine; CHMP: Committee for Medicinal Products for Human Use; CQ: Chloroquine; EMA: European Medicines Agency; GMP: Global Malaria Programme; ICH-GCP: International Conference on Harmonisation-Good Clinical Practice; IPTp: Intermittent preventive therapy in pregnancy; LBW: Low birth weight; MiP: Malaria in pregnancy; MMV: Medicines for Malaria Venture; SP: Sulphadoxine-pyrimethamine; STI: Sexually transmitted infection; WHO: World Health Organization

## Competing interests

RSC, JO, JR and PV are full-time employees of and own shares in Pfizer Inc. DU and SD are full-time employees of the Medicines for Malaria Venture.

## Authors’ contributions

All authors contributed to the trial protocol development. RC prepared manuscript outline and sections of first draft, reviewed and revised manuscript drafts, and approved the final version. All other authors reviewed and revised manuscript drafts, and approved the final version. All authors read and approved the final manuscript.
